# The concordance of European and US definitions for healthcare-associated infections (HAI)

**DOI:** 10.1186/1753-6561-5-S6-O2

**Published:** 2011-06-29

**Authors:** S Hansen, D Sohr, C Geffers, P Astagneau, A Blacky, W Koller, I Morales, ML Moro, M Palomar, E Szilagyi, C Suetens, P Gastmeier

**Affiliations:** 1 Charité - University Medicine Berlin, Berlin, Germany; 2 Université Pierre & Marie Curie, Paris, France; 3 Medical University Vienna, Vienna, Austria; 4 Scientific Institute of Public Health, Brussels, Belgium; 5 Agenzia Sanitaria e Sociale Regione Emilia Romagna, Bologna, Italy; 6 Hospital Vall d'Hebron, Barcelona, Spain; 7 National Centre for Epidemiology, Budapest, Hungary; 8 ECDC, Stockholm, Sweden

## Introduction / objectives

In Europe comparison of infection rates of HAI is restricted since some countries are using CDC definitions while others use HELICS (Hospitals in Europe Link for Infection Control through Surveillance) definitions. As part of the harmonization process of surveillance, ECDC outsourced a study to analyze the concordance between the definitions.

## Methods

A group with experts from 7 European countries was set up to realize the study. Agreement for bloodstream infection (BSI) and pneumonia (PN) was estimated by Cohens kappa.

## Results

The study was performed on 47 ICUs and 6506 patients, 180 PN and 123 BSI cases. Agreement for PN was k= 0.99 (CI95 0.98;1.00). When PN cases were divided in clinically and microbiologically defined PN, kappa values were 0.90 (CI95 0.86;0.94) and 0.72 (CI95 0.63;0.82) respectively. Diagnosis of PN varied among countries: 4 countries predominantly surveyed microbiologically defined PN whereas the others recorded mainly clinically defined PN. Agreement for BSI was k= 0.73 (CI95 0.66;0.80), BSI cases secondary to another infection site (42% of all BSI) were missed by CDC definitions. BSI concordance was perfect (k= 1.00) when only primary BSI cases (HELICS BSI with origin “catheter” or “unknown” and CDC BSI) were analyzed.

## Conclusion

Although other methodological differences exist between the two protocols, case definitions per se do not compromise comparability of results and should not be an obstacle for harmonization of European surveillance.

## Disclosure of interest

None declared.

